# Impaired osteoblast and osteoclast function characterize the osteoporosis of Snyder - Robinson syndrome

**DOI:** 10.1186/s13023-015-0235-8

**Published:** 2015-03-07

**Authors:** Jessica S Albert, Nisan Bhattacharyya, Lynne A Wolfe, William P Bone, Valerie Maduro, John Accardi, David R Adams, Charles E Schwartz, Joy Norris, Tim Wood, Rachel I Gafni, Michael T Collins, Laura L Tosi, Thomas C Markello, William A Gahl, Cornelius F Boerkoel

**Affiliations:** Undiagnosed Diseases Program, Common Fund, Office of the Director, National Institutes of Health, Bethesda, MD 20814 USA; Medical Genetics Branch, National Human Genome Research Institute, Bethesda, MD USA; Skeletal Clinical Studies Unit, Craniofacial and Skeletal Disease Branch, National Institute of Dental and Craniofacial Research, National Institutes of Health, Bethesda, MD 20892 USA; J.C. Self Research Institute, Greenwood Genetics Centre, Greenwood, SC 29646 USA; George Washington University School of Medicine, Washington, DC USA; Children’s National Medical Center, Washington, DC USA

**Keywords:** Spermine, Snyder-Robinson syndrome, Osteoblast, Osteoclast, Osteoporosis

## Abstract

**Background:**

Snyder-Robinson Syndrome (SRS) is an X-linked intellectual disability disorder also characterized by osteoporosis, scoliosis, and dysmorphic facial features. It is caused by mutations in *SMS*, a ubiquitously expressed gene encoding the polyamine biosynthetic enzyme spermine synthase. We hypothesized that the tissue specificity of SRS arises from differential sensitivity to spermidine toxicity or spermine deficiency.

**Methods:**

We performed detailed clinical, endocrine, histopathologic, and morphometric studies on two affected brothers with a spermine synthase loss of function mutation (NM_004595.4:c.443A > G, p.Gln148Arg). We also measured spermine and spermidine levels in cultured human bone marrow stromal cells (hBMSCs) and fibroblasts using the Biochrom 30 polyamine protocol and assessed the osteogenic potential of hBMSCs.

**Results:**

In addition to the known tissue-specific features of SRS, the propositi manifested retinal pigmentary changes, recurrent episodes of hyper- and hypoglycemia, nephrocalcinosis, renal cysts, and frequent respiratory infections. Bone histopathology and morphometry identified a profound depletion of osteoblasts and osteoclasts, absence of a trabecular meshwork, a low bone volume and a thin cortex. Comparison of cultured fibroblasts from affected and unaffected individuals showed relatively small changes in polyamine content, whereas comparison of cultured osteoblasts identified marked differences in spermidine and spermine content. Osteogenic differentiation of the SRS-derived hBMSCs identified a severe deficiency of calcium phosphate mineralization.

**Conclusions:**

Our findings support the hypothesis that cell specific alterations in polyamine metabolism contribute to the tissue specificity of SRS features, and that the low bone density arises from a failure of mineralization.

**Electronic supplementary material:**

The online version of this article (doi:10.1186/s13023-015-0235-8) contains supplementary material, which is available to authorized users.

## Background

Polyamines are ubiquitous, aliphatic, positively charged molecules that interact with anionic compounds such as DNA, RNA, and ATP [1,2]. Homeostasis of the polyamines putrescine, spermidine, and spermine is essential to cell growth and survival [3]. By addition of a propylamine moiety, spermidine synthase (SRM) converts putrescine into spermidine, and spermine synthase (SMS) converts spermidine into spermine [4]. The balance of spermine and spermidine is crucial for ion channel regulation, transcription and translation [5-9].

Mutations of *SMS*, the gene encoding spermine synthase, cause Snyder-Robinson syndrome (SRS), an X-linked disorder first reported in 1969 [10]. The clinical features of SRS include intellectual disability, dysmorphic facies, speech and gait abnormalities, seizures, muscle hypoplasia, kyphoscoliosis, and osteoporosis [11-17]. All affected males have hemizygous mutations in *SMS* that result in reduced SMS activity and a decreased spermine:spermidine ratio.

Atraumatic osteoporotic fractures commonly occur in individuals with SRS, leading to significantly impaired quality of life. Osteoporosis arises from disruption of the equilibrium between osteoclastic bone resorption and osteoblastic bone formation [18], which is regulated by mechanical and endocrine stimuli [19]. This general understanding of osteoporosis has led to established therapeutic interventions, but further insights are required to address the osteoporosis of SRS in a disease-specific manner.

Here we define the osteoporotic disease of SRS in two brothers with a missense mutation in *SMS* [20] and report depletion of osteoblasts and osteoclasts, reduced cancellous and cortical bone, reduced calcium-phosphate mineralization *in vitro*, and markedly abnormal polyamine content in human bone marrow stromal cells (hBMSCs). These data offer new insights into the role of polyamines in bone formation.

### Clinical reports

#### Patient II-1

**Figure 1:**
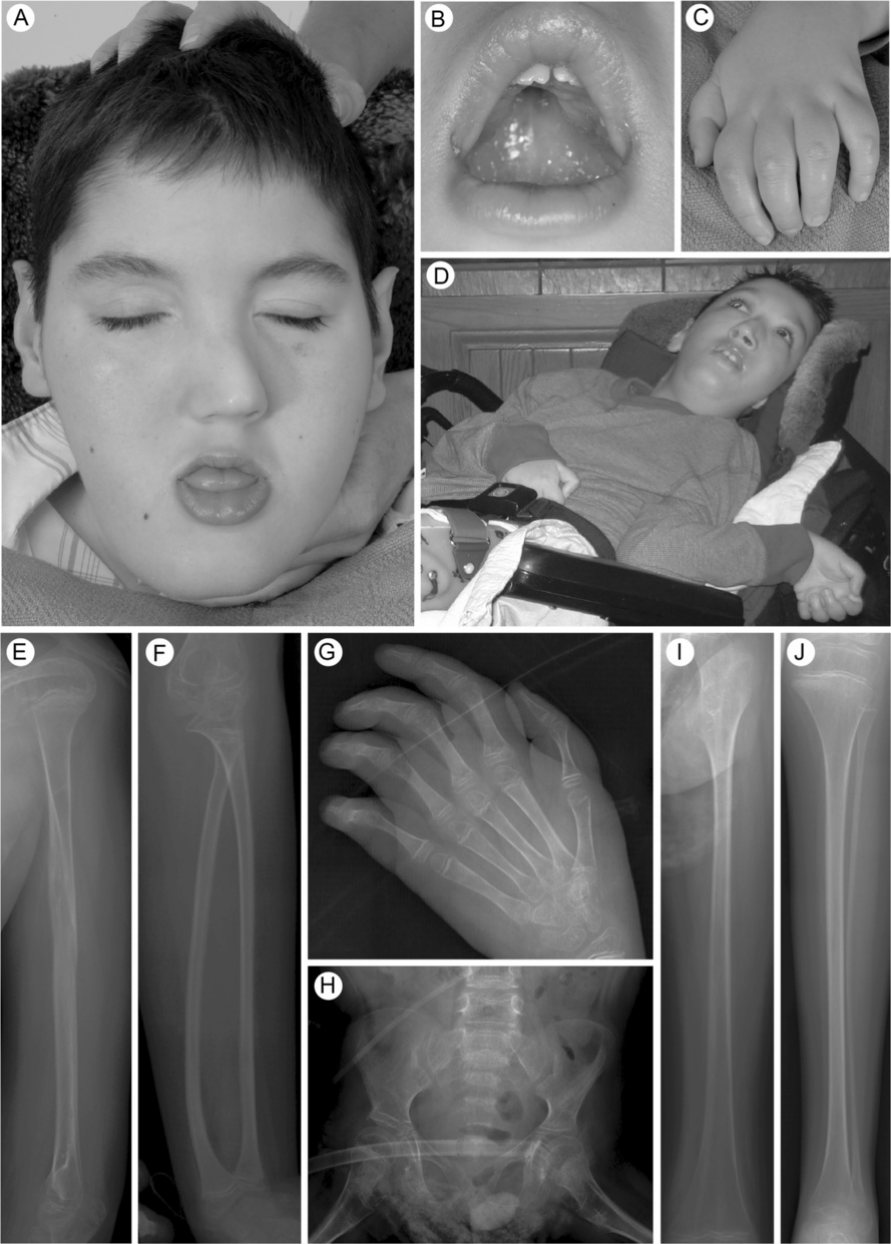
**Clinical and radiographic features. A**. Face of patient II-1. **B**. Face of patient II-3. **C**. Palate of patient II-1. **D**. Hands of patient II-3. **E**-**J**. Skeletal radiographs of Patient II-1 showing the left humerus **(E)**, left forearm **(F)**, left hand **(G)**, pelvis **(H)**, left femur **(I)** and left lower leg **(J)**. Note the gracile bones and undermineralization as well as the healing humeral fracture **(E)**.

The propositus (II-1, Figure 1) is the 18-year-old son of non-consanguineous healthy parents with no family history of intellectual disability or skeletal problems.

He was born by cesarean section at 40 weeks following a gestation complicated by poor maternal weight gain and, at 8 months, atypical fetal movements suggestive of *in utero* seizures. His birth weight, length and occipitofrontal circumference (OFC) were 2.38 kg (3%), 47 cm (13%) and 34.5 cm (26%), respectively. Apgar scores were 8 and 9 at one and five minutes. He had saggy skin but no other dysmorphic features. Following a perinatal intraventricular hemorrhage associated with thrombocytopenia (7×10^3^ cells/μl) that corrected after 3 platelet transfusions, he developed seizures, apnea with cyanosis, temperature instability and hypoglycemia. His recurrent episodes of hyperglycemia and hypoglycemia resolved with age and the placement of a gastric tube that allowed more frequent feedings.

Patient II-1 had tracheomalacia, upper airway obstruction, increased respiratory secretions, frequent aspirations, and pulmonary infections from infancy. By age 7 years, he had chronic *Pseudomonas aeruginosa* respiratory infection. At 6 years, he developed proximal renal tubular acidosis (RTA), nephrocalcinosis and nephrolithiasis. His renal stones were composed of carbonate apatite and calcium oxalate. His RTA has been managed with fluid and electrolyte replacement, spironolactone and hydrochlorothiazide.

Although initially controlled with phenobarbital, his seizures progressed to infantile spasms by 15 months. Adrenocorticotropic hormone (ACTH) treatment transiently reduced seizure frequency. His current anticonvulsant therapy includes rufinamide, felbamate, clonazepam, and topiramate. He had delayed development with regression of several developmental milestones. He smiled and laughed by 4 months, reached for toys by 6 months, had vocalizations by 7 months, and could hold his head up and roll onto his side by 8 months. He achieved his pincer grasp at 18 months but lost it by 23 months. He never walked. He lost many motor skills and all vocalization by 26 months of age. A cranial MRI at 14 years revealed cystic encephalomalacia and a low parenchymal T2 signal in the right temporal and right occipital lobes. These findings were considered consequences of the perinatal intraventricular hemorrhage.

When first evaluated at NIH at 15 years, Patient II-1 was awake but not interactive and could not sit independently or hold up his head; he withdrew from noxious stimuli. His height, weight and OFC were 129 cm (<3%ile), 30.8kg (<3%ile) and 50.3 cm (<3%ile), respectively. Facial dysmorphisms included a long, oval, asymmetric face, midface hypoplasia, down-slanting palpebral fissures, large, cupped ears, smooth philtrum, high-arched palate and prognathia (Figure 1). His dental enamel was hypoplastic and secondary teeth 2, 6, 10, and 11 were absent. He had excessive drooling, sluggish pupillary reflexes and left-sided hearing loss. He had frequent seizures, severe hypotonia, decreased muscle bulk, hypoactive deep tendon reflexes, kyphoscoliosis and flexion contractures of most large and small joints (Figure 1). He had one prepubertal (1-2 mL) testis that was undescended but palpable in the inguinal canal and one undescended testis, Tanner stage III pubic hair and a prepubertal phallus. His skin was remarkable for excretions of carbonate apatite, reflecting calcium/phosphate dysregulation. His ophthalmologic exam revealed retinitis pigmentosa and cortical blindness.

**Figure 2:**
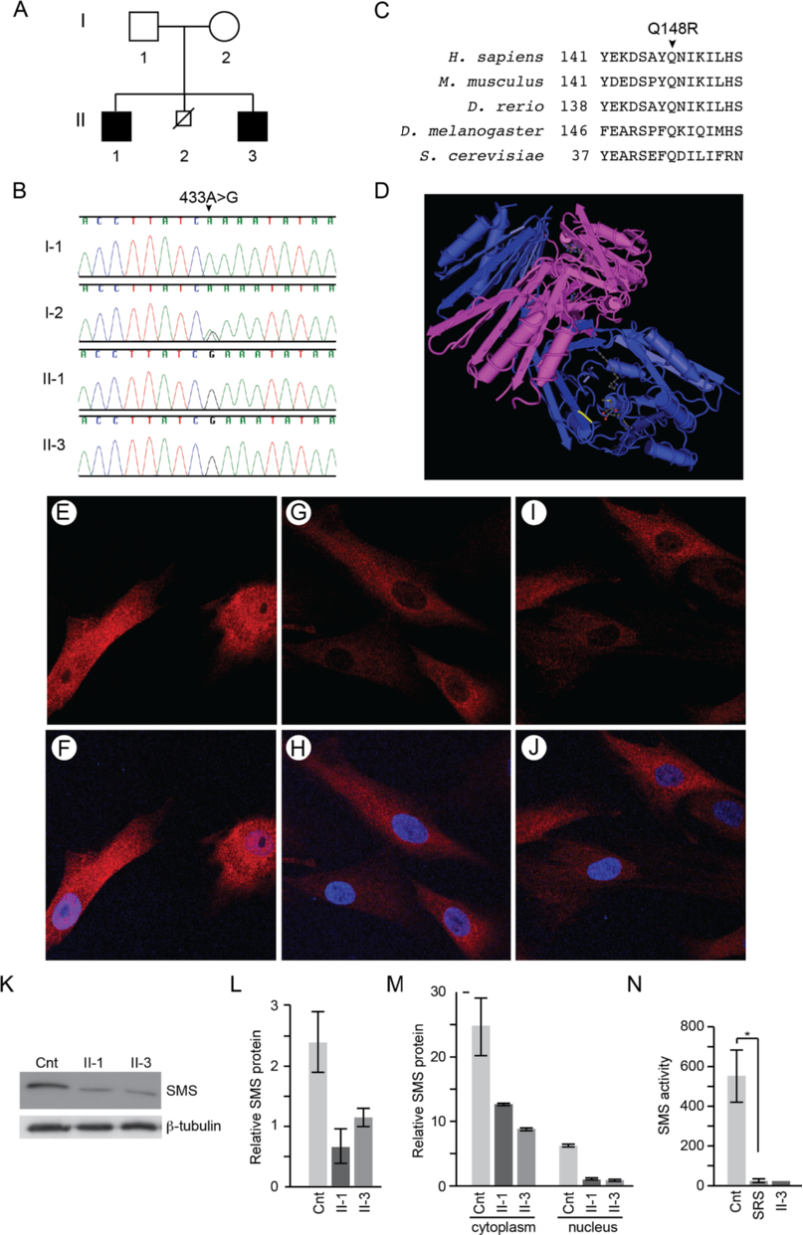
**Segregation, mutational analysis, and functional consequences of a novel** ***SMS*** **variant. A**. Pedigree of the family of the propositi. Affected males are shown by black squares. **B**. Sanger sequencing chromatograms showing the segregation of the *SMS* mutation NM_004595.4:c.443A > G from the carrier mother to the affected boys. The unaffected father did not have this mutation. **C**. Conservation of the p.Gln148 (p.Q148) residue across species. **D**. Drawing of the human SMS protein crystal complexed with spermidine and 5-methylthioadenosine. The mutated amino acid (Gln148) is highlighted in yellow [Mac PyMOL [23]]. **E**-**J**. Immunofluorescent detection of SMS protein subcellular distribution in unaffected **(E, F)**, Patient II-1 **(G, H)** and Patient II-3 **(I, J)** skin fibroblasts. SMS protein is shown in red and the nucleus is shown in blue. **K**. Immunoblot of skin fibroblast lysates showing reduced SMS protein levels in the patients (II-1, II-3) compared to an unaffected control (cnt). Tubulin is shown as a loading control. **L**. Graph showing steady state SMS protein levels in the patient and control fibroblasts relative to ß-tubulin levels. The data are based on 3 independent experiments for each cell line. **M**. Graph quantifying immunoblot detected steady state SMS protein levels in the cytoplasm and nuclei of patient and control fibroblasts. The cytoplasmic expression was normalized to β-tubulin expression and the nuclear expression to p84 expression. The data are based on 2 independent experiments for each cell line. **N**. SMS enzyme activity (spermidine d8 peak per hour) in lymphoblasts of unaffected individuals (Cnt), a cohort of 4 individuals with SRS (SRS) and patient II-1, * p < 0.05.

Previous skeletal problems included congenital bilateral hip dislocation and fractures of his distal fibula (2 years), right humerus (5 years) and spine (6 years). Kyphoscoliosis developed between 6 and 12 years of age. A dual-energy x-ray absorptiometry (DEXA) scan performed at age 15 years demonstrated a bone density of 0.341 gm/cm^2^ BMD (height adjusted Z-Score: -2.9 [21]) for the anteroposterior Spine (L1-L4) and 0.342 gm/cm^2^ bone mineral density (height adjusted Z-Score: -6.5 [21]) for the right forearm. Skeletal radiographs at 18 years revealed a 60° convex right scoliosis, gracile bones with reduced mineral density, and evidence of previous fractures (Figure 2E-J).

#### Patient II-3

Patient II-3, the brother of Patient II-1, was born at 37 weeks by cesarean section following an uncomplicated pregnancy. His birth weight was 2.81 kg (25%). During the immediate neonatal period his platelet count decreased from 90 to 45 k/μL, and he was admitted to the neonatal intensive care unit for treatment with dexamethasone and a platelet transfusion. He also had transient hypoglycemia, poor feeding and sensitivity to light. By 4 days of life, his condition had stabilized and he was discharged. Over the subsequent months, he manifested moderate laryngomalacia, mild tracheobrochomalacia, severe torticollis and abnormally pigmented retinas. Like his brother, he has also had episodic hyper- and hypoglycemia. He was diagnosed with nephrocalcinosis at 1 year and RTA by 2 years. The composition of his renal stones was carbonate apatite and calcium oxalate.

Patient II-3 had global developmental delay, severe hypotonia, and regression of milestones; he lost vocalizations and most motor skills by 15 months. An EEG at 6 months showed generalized slowing and disorganization; at 18 months he manifested seizures and hypsarrhythmia. An MRI performed at 2 years of age showed a mild increase in ventricular size but no other abnormalities. There was no evidence of hemorrhage or malformation. Since age 5 years, he has required a vagal nerve stimulator and bi-pap for adequate respiration. He also had repeated *Pseudomonas aeruginosa* pulmonary infections. His infections are more frequent and severe than are those of his brother.

Patient II-3 has had multiple atraumatic fractures involving the clavicle, tibia, femur and humerus. He also had congenital bilateral hip dislocation and, by 4 years of age scoliosis.

Illness prevented Patient II-3 from traveling to the NIH for evaluation, but review of his medical records and photographs showed that he was alert and non-ambulatory at age 10 years. He had facial features similar to those of his brother (Figure 1), superficial skin excretions, hypotonia, large joint contractures and muscle atrophy.

### Laboratory studies

The propositi had extensive laboratory testing. This identified an elevated antibody titer to myelin basic protein and a mild intermittent anemia with low iron saturation (7%; normal, 15-62) and ferritin (20 mcg/L; normal, 26 -388) for Patient II-1 and an elevated blood lactate level of 7.8 mmol/L (0.5-2.2) and mild elevations of urine carnitine esters for Patient II-3. Testing for abnormalities in organic acids, amino acids, acylcarnitines, very long chain fatty acids, lysosomal enzymes, biotinidase and copper were unremarkable. Molecular testing of *MECP2*, mitochondrial DNA, a panel of lysosomal storage disease-associated genes and the X-Linked Mental Retardation 9 Gene Panel (Greenwood Clinic, 2008), which did not include *SMS* and *FRMPD4*, did not detect pathogenic mutations. Patient II-1 had a normal karyotype (46,XY), and clinical and research copy number variant analysis did not detect any pathogenic variants (Additional file 1: Table S1).

## Methods

### Patients

The propositi were accepted into the NIH Undiagnosed Diseases Program (UDP) and enrolled in clinical protocol 76-HG-0238, approved by the Institutional Review Board of the National Human Genome Research Institute. Their parents gave written, informed consent.

### CNV analysis

The NHGRI Genomics Core lab performed SNP determinations using the Illumina Bead Array Platform (HumanOmniExpress, Illumina Corp., San Diego, CA, USA). Genome-wide fluorescent intensities and genotype calls were analyzed using Bead Studio and Genome Studio (Illumina Corp.). Analysis of copy-number variations was performed using PennCNV software, [22] and visual inspection using Genome Studio version 2010v3 build37/hg19 [23].

### Exome sequence analysis

Genomic DNA was extracted from whole blood using the Gentra Puregene Blood kit (Qiagen, Valencia, CA) according to the manufacturer’s specifications. Exome sequencing and analysis were performed as described [24-26]. The potential pathogenicity of identified variants was predicted using CDPred, SIFT, PolyPhen2 [27-33] or the BLOSUM62 scoring matrix [34].

### Confirmatory sequencing

For the *SMS* variant, Qiagen HotStarTaq master mix (Qiagen, Valencia, CA) was used to amplify the putative variant and 200 flanking nucleotides using the primers 5′-TGTGGCTTTCTTTTGCACAC-3′ and 5′-TGCATCTCAAAAACCAGCAG-3′. Unincorporated primers and nucleotides were removed using ExoSAP-IT reagent (USB, Cleveland, OH, USA). Sanger capillary sequencing was used to sequence the PCR products (Macrogen, Rockville, MD), and the sequences were aligned and analyzed using Sequencher v.4.10.1 (Gene Codes, Ann Arbor, MI, USA). Mutation interpretation was conducted using Alamut 2.0 (Interactive Biosoftware, San Diego, CA, USA).

### Bone biopsy and histomorphometry

Patient II-1 was given two courses of demeclocycline prior to the biopsy so that dynamic histomorphometry, including measurement of bone turnover, could be performed [35]. A bicortical transiliac crest core biopsy was performed and immediately split. The sample was placed in 70% ethanol and sent to The Johns Hopkins School of Medicine for histomorphometry, which was performed as described [35].

### Western blot analysis

Protein was extracted from fibroblast and osteoblast cell lines with RIPA buffer (Thermo Scientific, Waltham, MA) or used directly from Clontech human protein library (Clontech Laboratories, Mountain View, CA). Lysates (30ug) were electrophoresed on a 4-10% SDS-polyacrylamide gel and transferred to a polyvinylidene fluoride (PVDF) membrane. Using gentle agitation, the membrane was blocked overnight at 4ºC with casein blocking buffer (Thermo Scientific, Waltham, MA) and 10% horse serum. Anti-SMS (1:1000) (Novus Biologicals, Littleton, CO), anti-β-tubulin (1:1000, AbCam, Cambridge, UK), and anti-GAPDH (1:1000, GeneTex, Irvine, CA) were used as primary antibodies. Horseradish peroxidase-conjugated secondary antibodies (1:10000, Bio-rad Laboratories, Hurcules, CA) were used to detect the primary antibodies. The antibody-enzyme complexes were detected by chemiluminescence using Amersham ECL western blotting detection reagent (GE Life Sciences, Pittsburgh, PA) or WesternSure Premium Chemiluminescent Substrate (LI-COR, Lincoln, NE) according to the manufacturer’s specifications. β-tubulin or GAPDH was detected as a loading control.

### Cellular fractionation

Cellular fractionation was performed using NE-PER Nuclear and Cytoplasmic Extraction Reagents (Thermo Scientific, Waltham, MA) per the manufacturer’s recommendations. Immunoblotting was performed as described above. β-tubulin and p84 (GeneTex, Irvine, CA) served as loading controls for the cytoplasmic and nuclear fractions, respectively.

### qRT-PCR analysis

RNA was extracted from cells using the RNeasy Mini Kit (Qiagen, Valencia, CA) per the manufacturer’s specifications. RNA was converted to cDNA using the VILO cDNA synthesis kit (Life Technologies, Grand Island, NY) per the manufacturer’s protocol. 100ng of cDNA was amplified using Sso Advanced SYBR Green supermix (Bio-Rad Laboratories, Richmond, CA) per the manufacturer’s specification. qRT-PCR for measuring *SMS* steady state mRNA levels was performed on the Bio-Rad CFX96 Real-Time system (Bio-Rad Laboratories, Richmond, CA) using primers 5′-gattggtgttgctggacctt-3′ and 5′-tgactcaattctttcattctttcct-3′. PCR was cycled 33 times and annealing temperature was 58 degrees, melt curve was incremented at 0.5 degrees from 65-95 degrees. mRNA levels were normalized to *GAPDH* mRNA levels, a house-keeping gene.

### Cell culture

Epstein-Barr virus (EBV)-transformed lymphoblast cells were cultured as described previously [36]. Fibroblast cells were cultured in Dulbecco’s Modified Eagle Medium (DMEM) supplemented with 4.5 g/L D-glucose, L-glutamine, sodium pyruvate (Life Technologies, Grand Island, NY), 10% fetal bovine serum (Life Technologies, Grand Island, NY) and 1 X antibiotic (Life Technologies, Grand Island, NY). Fibroblasts were grown at 37°C with 5% CO2.

Human bone marrow stromal cells (hBMSC) were isolated and grown in culture as previously described [37]. Briefly, bone biopsy samples from patient(s) were used as the starting material. Cells were grown in α-minimal essential medium (α-MEM) containing 20% fetal bovine serum (FBS, Atlanta Biologicals, Lawrenceville, GA, USA), L-glutamine (Glutamax, GIBCO, Carlsbad, CA, USA), and penicillin-streptomycin mix. When noted, cells were also grown in the above medium containing Dexamethasone (10-8 M dexamethasone and 10-4 M ascorbate). Cells were cryopreserved in α-MEM containing 50% FBS and 5% dimethyl sulphoxide (DMSO). Control hBMSC were obtained from the rib of a 51 year-old Caucasian male.

### Osteogenic differentiation assay

Control and experimental BMSCs were plated (6x104/12-well plate) in triplicate. Cells were kept either untreated (α-MEM containing 20% FBS, L-glutamine, antibiotics) or treated with an osteogenic differentiation media (α-MEM, 20% FBS, L-glutamine, antibiotics, 5x10-3M β-glycerophosphate, 1x10-4M Ascorbic Acid Phosphate and 1x10-8M dexamethasone). Media was changed every 3-4 days. After 18 days, cells were rinsed with HBSS (Hanks’ Balanced Salt Solution, Invitrogen, Grand Island, NY) and were fixed at room temperature using 4% paraformaldehyde solution in 1X phosphate buffered saline (PBS). Cells were washed with distilled water, and were stained with Alizarin Red solution at room temperature for 20 minutes. Finally, the cells were washed with distilled water for 6 times and were photographed.

### Spermine synthase activity assay

To test SMS activity in lymphoblastoid cell lines, we measured the production of deuterated spermine (spermine d8) from deuterated spermidine (spermidine d8). Briefly, cells were harvested by centrifugation and washed 2X with PBS and suspended in 50mM sodium phosphate buffer pH 7.2 with protease inhibitor (Sigma, St. Louis, MO). Samples were frozen at -80ºC. Upon thawing, the samples were subjected to 2 freeze-thaw cycles using an ethanol and dry ice bath. After centrifugation, protein in the supernatant was quantified using the Lowry assay. To test for SMS activity, 70 μg of protein was incubated with 0.1M sodium phosphate buffer 7.5, 10μM spermidine d8 (Sigma, St. Louis, MO), protease inhibitor (Sigma, St. Louis, MO), 100μM dcSAM, and 50uM 4-MCHA (Sigma, St. Louis, MO) in a total volume of 100 μL. Baseline reactions were stopped immediately with 100μL acetonitrile/0.1% formic acid; other samples were incubated at 37ºC for 24 h and then stopped by addition of acetonitrile/0.1% formic acid. Spermine d8 was quantified by LC/MS/MS. Enzymatic activity was represented as the area of the spermidine d8 peak per hour.

### Measurement of polyamine content

The spermine/spermidine ratio was determined in the lymphoblastoid cell lines using LC/MS/MS as described [36]. Fibroblast and hBMSC polyamines were measured using the Biochrom 30 polyamine protocol. Briefly, human fibroblasts and hBMSCs were cultured as described above. Cells were harvested and pelleted and polyamines were extracted with a volume of 10% PCA equivalent to 4-fold the weight of the cells in milligrams. After 1 h of incubation on ice, cells were centrifuged at 13,000 rpm for 10 min at 4 degrees. The polyamines were fractionated and quantitated on the Biochrom 30 using an ion exchange polyamine column, compatible with sodium chemistry.

### Immunofluorescent localization of SMS

Immunofluorescent detection of SMS was modified from a previously described protocol [38]. Briefly, 2 x 104 cells were grown overnight on a coverslip in a 6-well plate. The cells were fixed with 3.7% paraformaldehyde (PFA) for 25 min at room temperature and permeabilized with 0.1% Triton X-100, 2mg/ml BSA and 1mM NaN3 for 5 min at room temperature. All cells were blocked for 2 h with casein blocking buffer with 10% horse serum and then incubated at 4°C overnight with anti-SMS (1:100) (Sigma, St. Louis, MO) diluted in blocking buffer. They were then gently washed 3 times with PBS and incubated with secondary antibodies conjugated with Alexa 488 or Alexa 555 (2ug/ml, Life Technologies, Grand Island, NY) for 2 h at room temperature. Cells were then washed 3 times with PBS and mounted in Vectashield containing 4′, 6-diamidino-2-phenylindole (DAPI, Vector Laboratories, Burlington, ON, Canada). Images were acquired using a 63X Zeiss plan-apochromat oil, 1.4 NA, DIC objective lens on a Zeiss LSM 780 confocal microscope using Zen 2011 acquisition software.

### Alkaline phosphatase activity

Upon reaching confluence, hBMSCs were plated in 6- well plates (100,000 cells/well). After 10 days of growth in MEM Alpha with 20% FBS, the hBMSCs were stimulated with 10mM β-glycerophosphate (Sigma St. Louis, MO), 50μM ascorbic acid 2-phosphate (Sigma, St. Louis, MO) and 100nM Dexamethasone (Sigma, St. Louis, MO) for 5 days. Alkaline phosphatase activity was quantified using StemTAG Alkaline Phosphatase Colorimetric Kit (Cell Biolabs, San Diego, CA).

## Results

### Exome sequencing identifies a novel SMS mutation diagnostic of SRS

The propositi had a maternally inherited hemizygous transition (NM_004595.4:c.443A > G) in *SMS* identified by exome sequencing (Additional file 1: Table S2; full VCF file is available on request) and confirmed by Sanger sequencing (Figure 2A-B). This mutation encodes the missense mutation p.Gln148Arg (CDPred score: -9; SIFT score: 0; PolyPhen2: probably damaging (0.998, Sensitivity 0.27, Specificity 0.99)). The Gln148 residue is conserved to *S. cerevisiae* (Figure 2C) and resides in the central β-strand domain that functions as a cap for the carboxyl terminal catalytic domain. It is one of 8 residues involved in the binding of 5’-methylthioadenosine (MTA) (Figure 2D) [39,40], which functions as an amine acceptor.

Similar to other SRS-associated *SMS* mutations [11-17], the p.Gln148Arg variant decreased the steady state level of SMS protein detectable in cultured fibroblasts by immunofluorescence and immunoblotting (Figure 2E-K). It reduced total SMS steady state levels 2.6-fold (Figure 2L); nuclear SMS was reduced 5.8-fold and soluble cytosolic SMS 2.7- fold (Figure 2M).

Spermine d8 generation by lymphoblastoid lysates expressing p.Gln148Arg SMS was 37- fold less than that for unaffected controls (Figure 2N). Additionally, as measured by LC/MS/MS, the ratio of spermine: spermidine in the lymphoblastoid lysates was reduced 10-fold compared to unaffected controls.

The propositi had features previously not reported with SRS, including more profound intellectual disability, retinal pigmentary changes, renal dysfunction, frequent pulmonary infections and hyper- and hypoglycemia (Table 1). Although we hypothesized that rare mutations in other genes contributed to these features, only a maternally inherited X-linked variant in *FRMPD4* (c.583A > G, p.K195E) segregated with the disease and had a predicted pathogenicity of at least probably damaging in Polyphen [27]; SIFT and CDPred predict the variant as tolerated. *FRMPD4,* which has been associated with autism and schizophrenia [41], encodes a product that regulates dendritic spine morphogenesis [42]. No pathogenic mutations were observed in genes associated with retinal pigmentary changes.

**Table 1:** **Comparison of clinical features of all reported SRS patients**

***Features***	***Reported patients***	***Patient II-1***	***Patient II-3***
Cognitive impairment	15/15	+	+
Seizures	8/15	+	+
Myopia	2/13	+ *	+ *
High, narrow palate	3/13	+	+
Prominent lower lip	12/15	+	-
Speech abnormalities	15/15	+ **	+ **
Diminished body bulk	15/15	+	+
Kyphoscoliosis	13/15	+	+
Osteoporosis	11/11	+	+
Long finger/toes	12/15	+ ***	#
Unsteady gait	10/15	+ ****	+ ****
Renal abnormalities	3/13	+	+
Nephrocalcinosis^a^	0/13	+	+
Frequent infections	0/13	+	+
Retinal pigment changes	0/13	+	+
Hypo-/Hyper-glycemia	1/13	+	+
Muscle fiber abnormalities	1/13	+	+

#Not assessed.

*Partial blindness.

**No vocalizations.

***Contractures of fingers.

****Non-ambulatory.

^**a**^Note: Nephrocalcinosis in these patients previously recorded in GeneReviews [20].

The phenotype data used to generate this table was collected from [11-17].

### Bone formation is decreased in patient II-1

**Figure 3:**
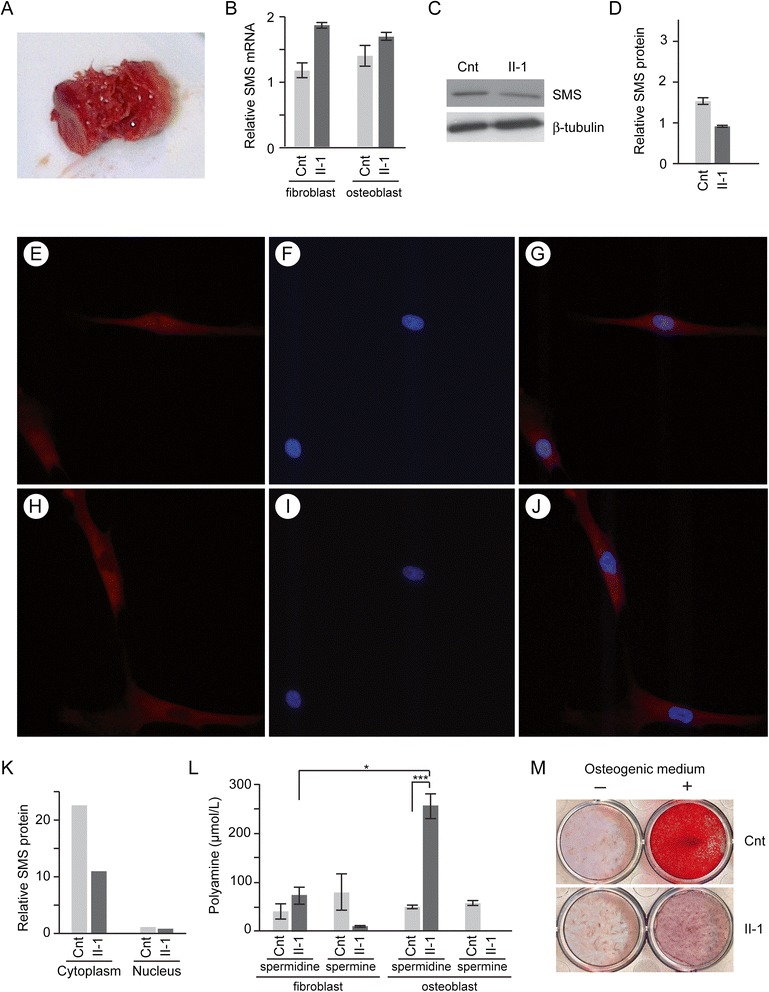
**Characterization of the bone and osteoblast pathology. A**. Photograph of the bone biopsy. **B**. Steady state SMS mRNA levels relative to GAPDH expression in cultured fibroblasts and osteoblasts. The patient’s cells did not differ significantly from controls. Data were derived by qRT-PCR analysis of 3 independent extractions of total RNA. **C**. Immunoblot showing steady state SMS protein expression in patient and control osteoblasts. ß-tubulin is shown as a loading control. **D**. Graph showing steady state SMS protein levels in the patient and control hBMSCs relative to ß-tubulin levels; there was no significant difference. The data are based on 3 independent experiments for each cell line. **E**-**J**. Immunofluorescent detection of SMS protein subcellular distribution in unaffected **(E-G)** and Patient II-1 **(H-J)** hBMSCs. SMS protein is shown in red and the nucleus is shown in blue. **K**. Graph quantifying immunoblot detected steady state SMS protein levels in the cytoplasm and nuclei of patient and control hBMSCs. The cytoplasmic expression was normalized to β-tubulin expression and the nuclear expression to p84 expression. **L**. Polyamine quantification in fibroblasts and osteoblasts. Note that the patient hBMSCs have a more striking imbalance of spermidine and spermine levels than do the patient fibroblasts, * p < 0.05, *** p < 0.005. **M**. Osteogenic potential of bone marrow stromal cells (hBMSCs) isolated from Patient II-1 sample is markedly lower than that of an unaffected control (cnt). The hBMSCs were seeded in triplicates (6x10^4^/12-well) and either kept untreated (-) or treated (+) with osteogenic differentiation media (see Methods) for 18 days. After the treatment, cells were fixed and were stained with Alizarin Red S to check for calcium deposition, a marker of osteogenic differentiation.

*SMS* is widely expressed (Additional file 1: Figure S1); consequently, we questioned why specific tissues such as bone are particularly affected in SRS patients. To define better the osteoporosis of SRS, we performed a bicortical transiliac crest core biopsy of Patient II-1. The specimen was soft and fragmented when removed from the trephine (Figure 3A). Histomorphometry revealed an absence of a trabecular meshwork, a low bone volume and a thin cortex. Cancellous bone volume was markedly decreased at 4.7%, compared with 23% ± 4.4 in healthy controls. The cortical mean width measured only 238 microns compared to a mean of 1202 microns in controls. Osteoblastic activity was markedly reduced with osteoblasts occupying only 1% of the osteoid surface (normal range 12.1% ± 4.6). There was no observable osteoclastic activity in the patient’s specimen, demonstrated by an eroded surface of 0% (normal range 4.1% ± 2.3) and an osteoclast surface of 0% (normal range 0.7% ± 0.6). Surface bone formation rate was 11.6 μm^3^/μm^2^/y (normal range: 35.8 μm^3^/μm^2^/y ± 8.9).

### CSF neurotransmitter levels do not suggest sympathetic nervous system dysfunction in Patient II-1

Because bone density and turnover are controlled in part by the sympathetic nervous system [43-49], we measured CSF neurotransmitters in Patient II-1 for evidence of increased sympathetic activity. The 5-methyltetrahydrofolate (methyl donor), 5-hydroxyindoleacetic acid (serotonin metabolite), 3-O-methyldopa (metabolite of L-Dopa), and tetrahydrobiopterin (cofactor for the synthesis of serotonin, dopamine, norepinephrine) were all within normal limits. Although homovanillic acid (catecholamine metabolite) (459 nmol/L) and neopterin (catabolic product of GTP) (30 nmol/L) were slightly above normal limits (324 and 28 nmol/L, respectively), these slight elevations were considered clinically insignificant.

### Endocrine evaluation in patient II-1

Because bone density is also modulated by multiple endocrine regulators [19], we assessed these in Patient II-1. Intact PTH, Vitamin D, magnesium, cortisol, insulin-like growth factor 1, thyroxine, thyroid stimulating hormone, prolactin and ACTH were unremarkable (Table 2). Also, manual inspection of the exome results did not identify any predicted deleterious mutations of *IGF1* or *IGF1R* despite >20 fold depth of short read coverage across the length of each exon. Prior to admission to the NIH, patients II-1 and II-3 had fluctuations in blood calcium and phosphate levels (Table 3). These fluctuations had no apparent pattern or mediating factor based on diet, fluid intake, or clinical status, and at the time of admission to NIH, the blood levels of calcium and phosphorus were normal. Tubular reabsorption of phosphate was normal, but, the calculated 24-hr urine calcium excretion (derived from an 18-hr collection) was elevated. Osteocalcin levels were low for pubertal stage, consistent with decreased bone formation. At age 15 years, patient II-1 had undescended testes, remarkably delayed puberty, low LH and FSH levels and undetectable testosterone. By age 18 years, he had entered early puberty as evidenced by an increase in LH and FSH, and testosterone levels in the Tanner II range. Testicular ultrasound at that time identified both testes in the inguinal canal, with volumes of 5.7 mL and 1.7 mL. At a chronological age of 18 years and 4 months, his hone age was 12 years and 6 months.

**Table 2:** **Markers of bone and endocrine function in Patient II-1 at 18 years of age**

***Metabolite***	***Patient II-1 values***	***Reference range***
Intact parathyroid hormone (pg/mL)	28.9	(15-65)
1,25-dihydroxycholecalciferol (pg/mL)	70	(18-64)
25-dihydroxycholecalciferol (ng/mL)	39	(33-100)
Osteocalcin (ng/mL)	35.7	(7.3-38.5)^1^, (49-167)^2^
Free thyroxine (ng/dL, direct dialysis)	1.6	(1-2.4)
Thyroid stimulating hormone (μIU/mL)	1.8	(0.4-4)
Testosterone (ng/mL)	43.6	(100-740)^1^, (8-418)^2^
Free testosterone (ng/dL)	0.5	(7.4-22.6)^1^
Sex hormone binding globulin (nmol/L)	63	(10-60)^1^,(44-160)^2^
Follicle stimulating hormone (U/L)	8.7	(1-11)^1^
Luteinizing hormone (U/L)	2.5	(1-8)^1^
Prolactin (mcg/dL)	9.4	(2-25)
Cortisol (mcg/dL, morning)	16.5	(5-25)
Adrenocorticotropic hormone (pg/mL)	12.7	(0.0-46.0)
Insulin-like growth factor (ng/mL)	347	(75-420)^2^
pH Urine	8.5	(5-8)
18-hr urine volume (mL/24 h)	2885	(600-1800)
Calculated urine calcium excretion (mmol/kg/24 h)	0.134	(<0.1)
Tubular reabsorption of phosphorus (%TRP)	93.5	(85-95)

^1^Reference ranges for age, ^2^Reference range for pubertal stage.

**Table 3:** **Calcium phosphate levels in patients II-1 and II-3**

***Serum Metabolite***	***Patient II-1***	***Patient II-3***	***Reference range***
Calcium (mg/dL)	7.8-10.6	6.6-10.1	8.78-10.5
Phosphorus (mg/dL)	2.4-5.1	3.5-5.3	3.1-5.1

### Compared to cultured SRS fibroblasts, SRS hBMSC have comparable SMS mRNA and protein levels but pronounced disturbances of polyamine levels

While absolute polyamine levels are not substantially abnormal in cultured fibroblasts and lymphoblastoid cells of SRS individuals, spermidine/spermine ratios reflected the SMS enzyme deficiency [12-17]. Neither lymphoblastoid cells nor skin fibroblasts, however, are affected tissues in SRS. We hypothesized therefore that cells from affected tissues such as bone had differences in SMS expression or polyamine metabolism accounting for their clinical manifestations. To test this, we compared SMS expression and polyamine levels among cultured skin fibroblasts and hBMSC. Cultured skin fibroblasts and hBMSC had comparable steady state SMS mRNA levels (Figure 3B) and SMS protein levels (data not shown), suggesting that differential SMS expression is an unlikely basis for expression of SRS features. Additionally, excluding differential degradation of the mutant SMS protein as the basis for expression of SRS features, the cultured hBMSC derived from Patient II-1 had SMS protein levels only 1.7-fold lower than for control hBMSC (Figure 3C-K), whereas his fibroblasts had 2.6-fold less SMS protein compared to control fibroblasts.

To test if differences in polyamine metabolism might contribute to the differential expression of SRS features, we compared polyamine levels in cultured skin fibroblasts and hBMSCs. Skin fibroblast lysates derived from Patients II-1 and II-3 had mean spermidine levels 1.78-fold higher than control fibroblasts (p = 0.28) and mean spermine levels 7.40-fold lower than control fibroblasts (p = 0.21). In contrast, lysates of cultured hBMSCs from Patient II-1 contained mean spermidine levels 5.07-fold higher (SEM = 22.8, p = 0.001) than control hBMSCs (Figure 3L) and had no detectable spermine.

### SRS hBMSCs have decreased osteogenic activity

To determine the differences in osteogenic potential, hBMSCs isolated from an unaffected control and Patient II-1 were treated with osteogenic differentiation media and stained with Alizarin Red S, an anthraquinone dye that stains the calcium deposits indicative of mature osteocytes [50,51]. Based on the intensity of Alizarin Red S staining, the differentiated hBMSCs from Patient II-1 produced markedly fewer calcium deposits than did those from the control (Figure 3M). Addition of 1μM spermine did not alter the Alizarin Red S staining (data not shown).

## Discussion

We report two brothers with SRS in whom we identified a *SMS* mutation (NM_004595.4:c.443A > G, p.Gln148Arg) resulting in near absence of enzyme activity and decreased steady state SMS protein levels. To better delineate the tissue-specificity of SRS features, we investigated the low bone density of SMS and observed functional osteoblast and osteoclast deficiencies, a marked spermidine and spermine imbalance in hBMSCs and poor calcium phosphate mineralization by differentiated hBMSCs. We therefore speculate that polyamines play a critical role in osteoblasts that is not required by other cells, such as lymphoblasts and fibroblasts.

The p.Gln148Arg mutation represents the first *SMS* mutation to alter the MTA binding site [52]. Disturbance of MTA homeostasis could contribute to the more severe phenotype of the propositi relative to that of other SRS patients. MTA is needed for the transfer of the aminopropyl group from decarboxylated S-adenosylmethionine (dcAdoMet) to spermidine; it is also an inhibitor of SRM and SMS [53], and consequently decreases cellular spermine concentrations [54]. Indeed, the spermine:spermidine ratio of our patients’ cells was reduced 3-fold more than the ratio for cells tested from any other SRS patient (C.S., unpublished data).

The severity of the phenotype in the propositi relative to other individuals with SRS might alternatively be attributable to differing insults or genetic backgrounds. For example, the perinatal intraventricular hemorrhage of patient II-1 might contribute to the severity of his neurological features, although the presence of similar neurological features in his brother, who did not have an ischemic or hemorrhagic brain insult, suggests this is not a substantial contributor. On the other hand, the variant in *FRMPD4* might modify the genetic background and thereby contribute to the intellectual disability of the propositi. We did not identify other environmental insults or appropriately segregating, pathogenic variants to explain the additional features of the propositi. Consequently, if other genetic contributors have a significant role in modifying the phenotype of SRS, they were either not detected by our exome sequencing or were common polymorphisms excluded by our analyses. It remains possible that epigenetic and stochastic factors also modulate the expressivity of SRS.

Central neuroendocrine signaling does not appear to be impaired in SRS; however, peripheral neuroendocrine signaling including hypogonadism and altered calcium and phosphate homeostasis might contribute as might other issues such as immobilization, renal tubular acidosis, low muscle mass and medications. It is thought that male hypogonadism decreases bone mineral density because androgens promote osteoblast differentiation and proliferation and decrease the activity of osteoclasts [55]. Additionally, impaired calcium and phosphate homeostasis impede osteoclast function [56], and hypercalciuria is associated with decreased bone density, as well as the nephrocalcinosis seen in this patient. Several anticonvulsants, including clonazepam and topiramate, are also associated with decreases in bone mineral density [57]. These factors alone are unlikely to fully account for the decreased bone mineral density observed in our patients, since other SRS individuals have had low bone density in the absence of these issues [11]. Rather, the predominant mechanism of osteoporosis in SRS is likely related to impaired polyamine metabolism.

The disease mechanisms and phenotypic expansion reported herein provide some insight for the management of SRS. Optimal control of the metabolic abnormalities, limited use of medications known to affect bone, and appropriate physical therapy should be part of the management plan in any chronically ill, immobilized individual. Since analysis of the bone did not detect increased osteoclastic activity, bisphosphonate therapy would likely be of minimal effectiveness unless further studies refute our observations. In addition, if study of additional patients establishes the association of renal disease, retinal pigmentary changes, and perturbations of glucose homeostasis with SRS, then screening for and symptomatic management of these problems has the potential to improve patient care.

## Conclusions

This report identifies a novel SRS-associated *SMS* mutation, p. Gln148Arg, and expands the SRS phenotype. It also provides the first evidence that SRS patients have a loss of osteoblast and osteoclast activity and that the low bone density of SRS likely arises by a cell intrinsic process.

## Consent

The patients’ parents gave written, informed consent for publication of this case report and any accompanying photographs.

## Additional file

Additional file 1: Table S1. CNVs detected in the propositi. **Table S2.** Exome variants meeting rarity and predicted deleteriousness requirements and segregating with disease. **Figure S1.** Expression profile of SMS mRNA and protein. (A) Graph showing qRT-PCR detection of SMS mRNA levels in total RNA extracted from the respective tissues. SMS mRNA levels were normalized to the mRNA levels of GAPDH. (B) Immunoblot showing SMS protein levels in lysates from the respective tissues. (C) SMS protein expression in each tissue plotted relative to GAPDH protein expression.
