# Supplementary material for: Impaired osteoblast and osteoclast function characterize the osteoporosis of Snyder - Robinson syndrome
Source: Orphanet J Rare Dis. 2015 Mar 7;10:27. doi: 10.1186/s13023-015-0235-8 (PMC4428506; doi:10.1186/s13023-015-0235-8)
Supplement: Additional file 1: Table S1. — CNVs detected in the propositi. Table S2. Exome variants meeting rarity and predicted deleteriousness requirements and segregating with disease. Figure S1. Expression profile of SMS mRNA and protein. (A) Graph showing qRT-PCR detection of SMS mRNA levels in total RNA extracted from the respective tissues. SMS mRNA levels were normalized to the mRNA levels of GAPDH. (B) Immunoblot showing SMS protein levels in lysates from the respective tissues. (C) SMS protein expression in each tissue plotted relative to GAPDH protein expression. [file 13023_2015_235_MOESM1_ESM.pdf]

Supplementary Table 1. CNVs detected in the propositi.

UDP_929				UDP_930				Interpretation
CNV region (hg19)	Copy number	Parent of origin	No. of RefSeq genes affected	CNV region (hg19)	Copy number	Parent of origin	No. of RefSeq genes affected	
chr02:242873800-243034519	1	Both	3	chr02:242873800-243034519	1	Both	3	Common deletion-unlikely pathogenic
chr03:65191847-65214533	1	Maternal	0	chr03:65191847-65214533	1	Maternal	0	Common deletion-unlikely pathogenic
chr03:162131408-162142475	1	Paternal	0					Common deletion-unlikely pathogenic
chr04:64138341-64150666	1	Paternal	0					Common deletion-unlikely pathogenic
chr04:161059762-161071059	1	Paternal	0	chr04:161059762-161071059	1	Paternal	0	Common deletion-unlikely pathogenic
				chr05:97048466-97099320	1	Maternal	0	Common deletion-unlikely pathogenic
				chr05:140225908-140233387	1	Maternal	9	Common deletion-unlikely pathogenic
chr06:29855945-29881038	1	Maternal	3					Common deletion-unlikely pathogenic
chr06:32282216-32288238	1	Paternal	1					Father unaffected-unlikely pathogenic
chr07:40440233-40476625	1	Maternal	1	chr07:40440233-40476625	1	Maternal	1	Mother unaffected-unlikely pathogenic
chr07:157746669-157803711	3	Paternal	1					Father unaffected-unlikely pathogenic
chr10:59172666-59379460	3	Maternal	0	chr10:59172666-59379460	3	Maternal	0	Mother unaffected-unlikely pathogenic
chr11:84350040-84574040	1	Paternal	1	chr11:84350040-84574040	1	Paternal	1	Father unaffected-unlikely pathogenic
				chr11:8959020-8964938	1	Maternal	0	Mother unaffected-unlikely pathogenic
				chr12:27648411-27654546	1	Paternal	1	Father unaffected-unlikely pathogenic
chr13:69247022-	1	Maternal	0	chr13:69247022-	1	Maternal	0	Common deletion-

69267981				69267981				unlikely pathogenic
				chr16:87709933-87714284	1	Paternal	1	Father unaffected-unlikely pathogenic
				chr18:13798739-13804919	1	Maternal	0	Mother unaffected-unlikely pathogenic
chr18:66747568-66755508	1	Maternal	0					Common deletion-unlikely pathogenic
chr18:67209141-67217271	1	Maternal	0					Common deletion-unlikely pathogenic

Supplemental Table 2. Exome variants meeting rarity and predicted deleteriousness requirements and segregating with disease.

Chr	Position (hg19)	Ref Allele	Var Allele	Gene	Transcript	Protein change	dbSNP	Phenotype_Score	Variant_Score	Exomiser_Score	Genotype	Sanger	Seg
17	4795537	C	G	MINK1	NM_153827.4	intron 17		0.33	0	3.75E-05	Het	C/G	Y
17	4796267	C	T	MINK1	NM_153827.4	intron 19	rs142172345	0.33	0	3.75E-05	Het	C/T	Y
X	21995292	A	G	SMS	NM_004595.3	p.Gln148Arg	rs397515551	0.73	1	9.30E-01	Hemi	G	Y
X	12704225	A	G	FRMPD4	NM_014728.3	p.Lys195Glu	rs146493613	0.12	0.99	9.41E-03	Hemi	G	Y
01	989899	C	T	AGRN	NM_198576.2	p.Thr1983Met	rs147569326	0.30	0.86	2.63E-02	Het	C/T	N
01	10753989	G	A	CASZ1	NM_001079843.1	intron 3	rs185185302	0	0	8.62E-07	Het	*	*
01	204125492	-	CAGACAGA	REN	NM_000537.3	intron 7		0.73	0	4.10E-03	Het	-/CAGACAGA	N
01	180910200	A	G	KIAA1614	NM_020950.1	p.Thr980Ala	rs201945338	0.55	0	5.26E-04	Het	A/G	N
02	97851096	A	C	ANKRD36	NM_001164315.1	intron 29		0	0	8.62E-07	Het	A/A	N
03	47037984	G	A	NBEAL2	NM_015175.2	p.Arg792Gln	rs140354422	0.63	0.84	5.06E-01	Het	*	*
03	49723739	A	C	MST1	NM_020998.3	intron 8		0.28	0.60	2.75E-03	Het	A/A	N
04	5746919	C	T	EVC	NM_153717.2	intron 6	rs61514191	0.74	0	4.71E-03	Het	TTC/-	N
07	141537945	T	A	PRSS37	NM_001008270.2	intron 2	rs11766230	0.20	0	8.58E-06	Het	A/T	N
08	10470571	G	A	RP1L1	NM_178857.5	p.Ala346Gly		0.75	1	9.39E-01	Het	G/A	N
10	17164788	A	-	CUBN	NM_001081.3	intron 6		0.28	0	2.33E-05	Het	A/-	N
11	92573911	G	A	FAT3	NM_001008781.2	p.Val3518Met	rs10765565	0.44	0.79	7.20E-02	Het	T/A	N
12	6472752	G	A	SCNN1A	NM_001159576.1	p.Arg240Gly	rs55797039	0.73	0.29	3.85E-02	Het	GC/AA	N
15	77087795	T	C	SCAPER	NM_020843.2	intron 8	rs189913101	0	0	8.62E-07	Het	T/C	N
16	14014242	T	A	ERCC4	NM_005236.2	intron 1		0.77	0	6.31E-03	Het	T/A	N

16	84213684	C	T	TAF1C	NM_005679.3	p.Gly523Arg	rs4150167	0.62	0.15	3.57E-03	Het	C/T	N
17	14139702	G	A	CDRT15	NM_001007530.1	p.Ala103Gly	rs138924466	0.52	0	3.60E-04	Het	G/A	N
18	22642716	C	A	ZNF521	NM_015461.2	intron 7	rs199507114	0.27	0	2.07E-05	Het	*	*
19	1581272	C	T	MBD3	NM_001281453.1	intron 4	rs142198786	0.59	0	7.66E-04	Het	C/T	N
19	19646467	G	A	YJEFN3	NM_198537.3	p.Val225Met	rs200227282	0	0.81	5.85E-04	Het	G/A	N
19	42853636	TCAC	-	MEGF8	NM_001271938.1	intron 13		0.33	0	3.78E-05	Het	TCAC/-	N

Abbreviations: Chr, chromosome; Ref, reference; Sanger, Sanger sequencing result; Seg, segregation with disease in family; Var, variant

*Variants not confirmed by Sanger sequencing because the variants lay in repetitive sequence that could not be sequenced by standard PCR amplification of genomic DNA and dideoxy terminator sequencing methods.

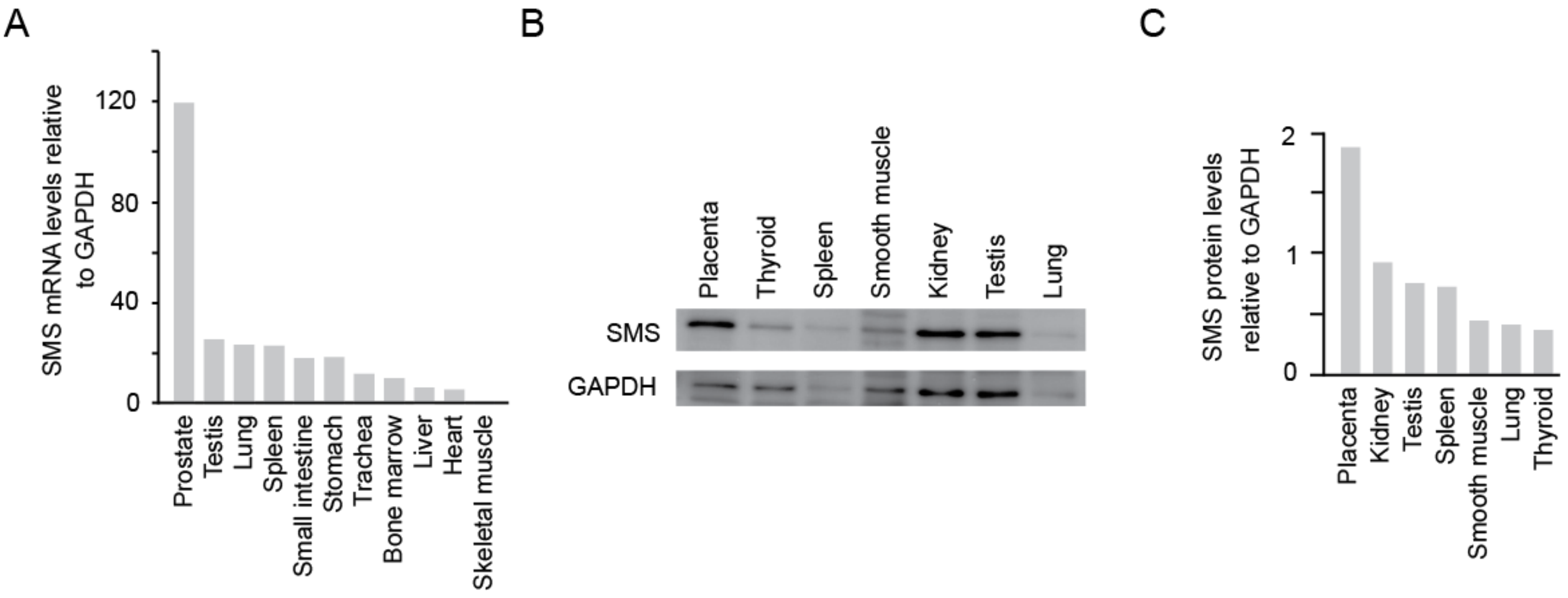

Supplementary Figure 1: Expression profile of SMS mRNA and protein. (A) Graph showing qRT-PCR detection of SMS mRNA levels in total RNA extracted from the respective tissues. SMS mRNA levels were normalized to the mRNA levels of GAPDH. (B) Immunoblot showing SMS protein levels in lysates from the respective tissues. (C) SMS protein expression in each tissue plotted relative to GAPDH protein expression.
